# Genome-Wide Identification and Expression Analysis of the NHX (Sodium/Hydrogen Antiporter) Gene Family in Cotton

**DOI:** 10.3389/fgene.2020.00964

**Published:** 2020-08-18

**Authors:** Xiaokang Fu, Zhengying Lu, Hengling Wei, Jingjing Zhang, Xu Yang, Aimin Wu, Liang Ma, Meng Kang, Jianhua Lu, Hantao Wang, Shuxun Yu

**Affiliations:** ^1^State Key Laboratory of Cotton Biology, Institute of Cotton Research, Chinese Academy of Agricultural Sciences, Anyang, China; ^2^Handan Academy of Agricultural Sciences, Handan, China

**Keywords:** *Gossypium*, Na^+^/H^+^ antiporter protein, gene expansion, salt tolerance, stress response

## Abstract

The sodium/hydrogen antiporter (NHX) gene family with the Na^+^/H^+^ exchange protein domain is a transporter of sodium and hydrogen ions and plays an important role in the response of plants to salt stress. Studying the response of cotton to salt stress through comprehensive identification and analysis of NHX genes in several species and their roles in salt tolerance mechanisms is of great significance. In this study, 23, 24, 12, and 12 NHX genes were identified from *Gossypium hirsutum* (Gh), *G. barbadense*, *G. arboreum* and *G. raimondii*, respectively. Phylogenetic analysis showed that these genes were mainly divided into three clades with significant subcellular localization, namely, endosome (Endo-class), plasma membrane (PM-class) and vacuole (Vac-class). By analyzing the structure of NHX genes and proteins, each branch of the NHX gene family was found to be structurally conserved, and collinearity analysis showed that NHX genes were mainly expressed through whole genome and segmental duplication. The non-synonymous (Ka)/synonymous (Ks) values showed that the NHX gene family experienced strong purifying selection during long-term evolution. *Cis*-acting element analysis showed that the NHX gene family may be related to the regulation of abscisic acid (ABA) and methyl jasmonate (MeJA) hormones. Additionally, transcriptomic data analysis and qRT-PCR showed that GhNHXs exhibited different expression patterns in each tissue and under different salinities. These results provide an important reference for us to further understand and analyze the molecular regulation mechanism of cotton NHX genes.

## Introduction

Soil salinization is one of the limiting factors of global crop yield, and approximately 800 million serious salinization events occur worldwide (Rengasamy, 2006; Munns and Tester, 2008). Increasingly serious soil salinization is an urgent problem for China’s agriculture (Flowers, 1999; Tomoaki and Julian, 2004; Heng et al., 2012; Deinlein et al., 2014). Salt stress can seriously affect plant growth and development and other normal life activities, resulting in the loss of crop yield and quality (Sun et al., 2019). Therefore, mining salt-tolerant genes, studying their functions through molecular analysis, and improving crop salt tolerance through molecular breeding are of great significance.

Plants activate a series of coping mechanisms when under salt stress, such as the use of hormones to regulate plant growth and metabolism, ion homeostasis and osmotic regulation (Wu G. Q. et al., 2019). Previous studies have found that the sodium/hydrogen antiporter (NHX) protein is key to the regulation mechanism of ion homeostasis (Pehlivan et al., 2016; Cao et al., 2020). The NHX genes mainly use two proton pumps formed by the H^+^-ATP enzyme and H^+^-PPase to produce H^+^ electrochemical gradients to transport Na^+^ from the cytoplasm to vacuoles or outside the cell, thereby maintaining the stability of Na^+^ ions and avoiding the toxic effect of Na^+^ accumulation in cells (Munns, 2002; Zorb et al., 2005; Sze and Chanroj, 2018). NHX proteins are ubiquitous transmembrane proteins belonging to the monovalent cation/proton antiporter 1 (CPA1) superfamily (Brett et al., 2005; Chanroj et al., 2012), and most NHX proteins contain 10–12 transmembrane helix domains (TMs) (Yamaguchi et al., 2003; Wu G. Q. et al., 2019). The NHX proteins are mainly localized in vacuoles, endosomes and plasma membranes (Aharon et al., 2003; Pehlivan et al., 2016). The first plant NHX gene was identified by Roberto et al. (1999) in *Arabidopsis thaliana* (At) (Roberto et al., 1999), and a total of 8 NHXs have been identified in this species to date. Of these NHXs, 2 genes (*AtNHX7* and *AtNHX8*) are located in the PM-class (plasma membranes), 2 genes (*AtNHX5* and *AtNHX6*) are located in the Endo-class (endosomes), and 4 genes (*AtNHX1-4*) are located in the Vac-class (vacuoles) (Shi et al., 2000; Aharon et al., 2003; Brett et al., 2005; Bassil et al., 2011).

NHX genes are involved in the salt stress response, cell expansion, membrane vesicle trafficking, and pH homeostasis (Dragwidge et al., 2019; Jegadeeson et al., 2019). Developmental disorders and cell division abnormalities in embryo and root tissues were observed after double knockout of *AtNHX5* and *AtNHX6* in *A. thaliana* (Dragwidge et al., 2018). In rice, salt stress, hyperosmotic stress and abscisic acid (ABA) stress induced expression of *OsNHX1*, *OsNHX2*, *OsNHX3* and *OsNHX5*, and the differential expression of NHX genes in different tissues may be an important factor determining the salt tolerance of rice (Fukuda et al., 2011). Salt-tolerant *perennial ryegrass* was obtained by transforming the rice vacuolar membrane *OsNHX1* gene, and the plant was found to be able to survive under high salt conditions (350 mM) for 10 weeks (Wu et al., 2005). Transgenic *Brassica napus* plants overexpressing a vacuolar NHX gene were able to grow, flower, and produce seeds in the presence of 200 mM NaCl (Zhang et al., 2001). Vac-class membrane gene *AgNHX1* of *Atriplex gmelinii* was transferred into rice, and the transgenic plants could survive under conditions of 300 mM NaCl for 3 days, while the wild-type rice plants could not (Ohta et al., 2002). In addition, transfer of the *AtNHX1* gene from *A. thaliana* into tomato (Eduardo et al., 2009) and transfer of the Vac-class membrane gene *SsNHX1* (*Salsola collina Pall.*) into *Medicago sativa* (Li et al., 2011) revealed that all plants with high expression of receptors significantly improved their salt tolerance.

Cotton is an important economic crop, and with the gradual transfer of cotton agriculture to Xinjiang (China) and saline-alkali beaches, improving the salt tolerance and stress resistance of cotton is very important (Gao et al., 2018; Dong et al., 2019). Some studies have shown that under a high concentration of NaCl (200 mM), NHX genes may play an important role in the salt tolerance of cotton (Kumar et al., 2017; Elsawy et al., 2018; Mushke et al., 2019). For example, cotton overexpressing *AtNHX1* can produce more biomass and fiber (He et al., 2005), and the expression level of *GhSOS1* (a plasma membrane NHX gene) in cotton roots is significantly increased (Chen et al., 2017). However, at present, research on the number and salt tolerance of NHX genes in cotton is limited. Therefore, we selected four cotton species [*Gossypium arboretum* (Ga), *G. raimondii* (Gr), *G. hirsutum* (Gh) and *G. barbadense* (Gb)] as the research objects, and a total of 71 NHX genes were identified. Through systematic analysis of the gene distribution, gene structure, phylogenetic relationships and *cis*-acting elements of the NHX family, the purpose of this paper is to provide a theoretical reference for the study of salt tolerance and the improvement of new cotton varieties in the future.

## Results

### Chromosomal Distribution of NHXs in *Gossypium* spp.

**FIGURE 1:**
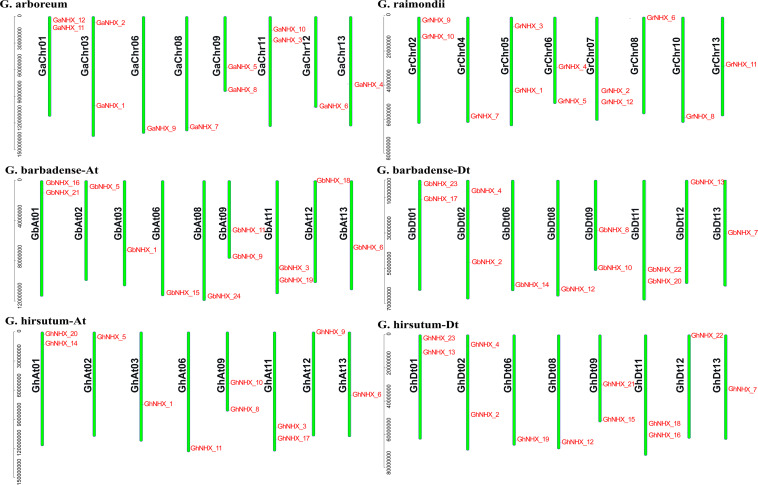
NHXs are distributed in the chromosomes of four cotton species (*G. arboreum*, *G. raimondii*, *G. barbadense*, and *G. hirsutum*), and the chromosome numbers are shown on the left side of each vertical bar.

A total of 23, 24, 12, and 12 NHX family genes were identified in *G. hirsutum, G. barbadense, G. raimondii*, and *G. arboreum*, respectively. The NHX genes were named according to their homology with *A. thaliana* genes from high to low (Supplementary Table S1). The position of each gene on the chromosome is shown in Figure 1. Through identification and analysis of the NHX genes of the four cotton species, their locations and distributions in chromosomes and the numbers of genes were found to be conserved in the evolution from diploid to tetraploid cotton species.

### Identification of the Physicochemical Properties of NHX Genes

The allotetraploid species *G. hirsutum* and *G. barbadense* have 11 and 12 NHX genes in the At subgenome, respectively, and 12 NHX genes in the Dt subgenome. Diploid species *G. arboreum* and *G. raimondii* each have 12 NHX genes (Figure 1). The transcripts of 71 genes are 963–7055 bp, and the protein sequences are 320–1166 aa. The isoelectric point (pI) of the protein is between 5.237 and 10.181, and the molecular weight is between 35.548 kDa and 129.861 kDa. Among 71 proteins, the number of TMs varies from 5 to 12, and that of 53 proteins ranges from 10 to 12 (Supplementary Table S2).

Through analysis of the number of phosphorylation sites of 75 protein sequences in *G. hirsutum*, *G. barbadense, G. arboreum*, and *G. raimondii*, 17 (*GhNHX_21*) – 76 (*GhNHX_1* and *GbNHX_1*) serine (S) and 4 (*GhNHX_20*) – 24 (*GhNHX_2* and *GbNHX_2*) threonine (Th) sites were found; *GhNHX_20* and *GbNHX_24* did not contain tyrosine (Ty), and *GhNHX_1, GhNHX2, GbNHX_1, GbNHX_2, GaNHX_1*, and *GrNHX_1* each contained 9 tyrosine sites, which was the highest number of sites. Among the three kinds of proteins, the content of serine was higher than those of threonine and tyrosine, indicating that serine is a common phosphorylation site in the upland cotton NHX family. In addition, the phosphorylation degrees of protein kinase C (PKC) and UNSP in NHX proteins were the highest, and the phosphorylation degrees of PKB, SRC, GSK3, and ATM were lower (Supplementary Table S3).

### Phylogenetic Analysis of NHX Sequences

**FIGURE 2:**
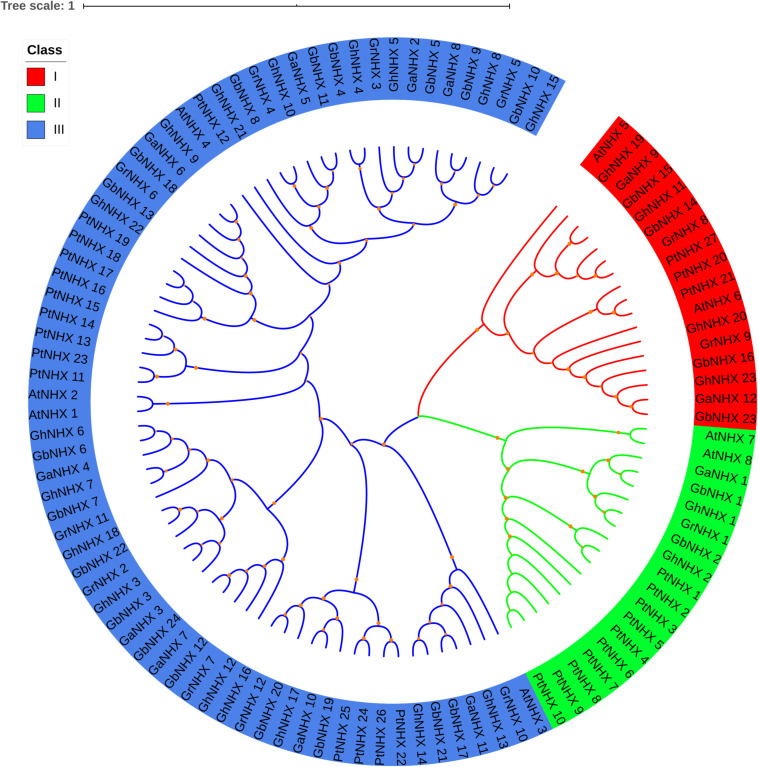
Phylogenetic tree of the NHX proteins in six species. Three categories of NHXs are indicated using different colors by iTOL.

To study the evolutionary relationships of the NHX gene family, a total of 106 NHX protein sequences from six species [*G. hirsutum*, *G. raimondii*, *G. barbadense*, *G. arboreum*, *Arabidopsis* and *Populus trichocarpa* (Pt)] were used to construct a phylogenetic tree (Figure 2). Based on the results of the phylogenetic tree combined with those of previous studies in *Arabidopsis*, 106 genes were mainly divided into three categories, namely, class I (Endo-class), class II (PM-class) and class III (Vac-class). Class III (Vac-class) was the largest group among the 6 species, which included 71 proteins (17 NHXs in *G. hirsutum*, 18 NHXs in *G. barbadense*, 9 NHXs in *G. arboreum*, 9 NHXs in *G. raimondii*, 4 NHXs in *Arabidopsis* and 14 NHXs in *P. trichocarpa*). Seventeen and 18 NHXs were distributed in class I (Endo-class) and class II (PM-class), respectively.

### Evolutionary Analysis of NHX Genes in *Gossypium* spp.

**FIGURE 3:**
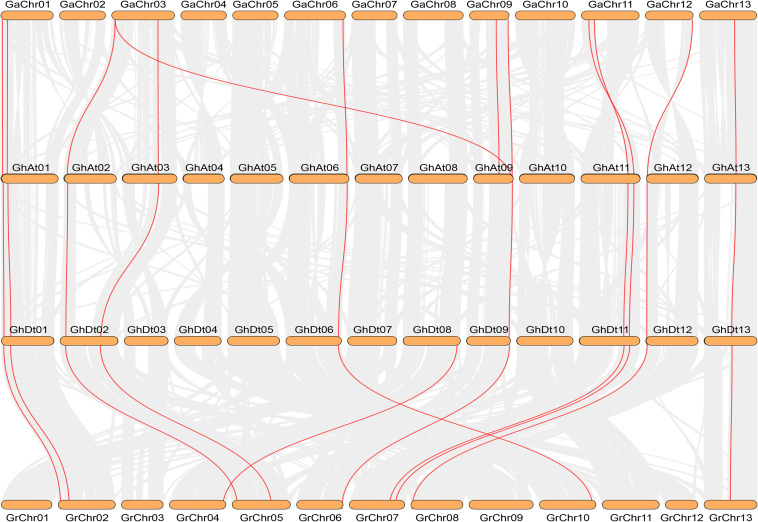
Collinearity analysis between *G. arboreum* (AA), *G. hirsutum* (At and Dt subgenomes) and *G. raimondii* (DD) by MCScanX. Red and gray lines depict NHX homologous gene pairs and WGD pairs, respectively.

The number of NHX genes in the two diploid cotton species, *G. raimondii* (12) and *G. arboreum* (12), was approximately half that in the tetraploid cotton *G. hirsutum* (23) and *G. barbadense* (24) (Figure 2). The NHX gene family was further verified to have emerged due to chromosome doubling during cotton evolution from diploid to tetraploid. Therefore, we speculate that during evolution, diploids may have become tetraploids through whole-genome duplication (WGD). To verify this hypothesis, we used MCScanX software to analyze the collinearity of the At and Dt subgenomes of *G. hirsutum* and the corresponding ancestral A and D diploid genomes. The results showed that the homologous gene pairs of GhNHX genes were in the collinearity analysis section between different genomes (Figure 3). These results provide strong evidence for the hypothesis that NHX genes were obtained by whole-genome duplication (WGD) or segmental duplication, and we analyzed the duplication types of the NHX genes in *G. hirsutum* and *G. barbadense*, as shown in Supplementary Table S4. The results showed that 46 of 47 NHXs were obtained by WGD or segmental duplication during evolution from diploid to tetraploid cotton species.

To study the selective pressure of NHX genes, homologous gene pairs were determined from the results of the gene sequence comparison program BLASTP and collinearity analysis. In this study, the homologous genes from diploid species to the heterotetraploid subgenome were mainly analyzed. A total of 44 pairs of homologous genes were identified by collinearity analysis between the allotetraploid (*G. barbadense* and *G. hirsutum*) genomes and the corresponding diploid genomes (*G. raimondii* and *G. arboreum*) (Figure 3 and Supplementary Table S5). Eleven homologous gene pairs each were identified in the *G. barbadense* and *G. hirsutum* subgenomes and the corresponding *G. arboreum* (Ga) genomes, except for *GaNHX_7*. The other 11 NHX genes all had homologous gene pairs in tetraploid cotton species, and 22 homologous gene pairs were found in *G. raimondii* and the corresponding *G. barbadense* and *G. hirsutum* Dt subgenomes. Among the 44 homologous gene pairs identified, the selection pressure of these gene pairs was evaluated by calculating non-synonymous (Ka)/synonymous (Ks) values, and 42 pairs had a Ka/Ks ratio < 1 (Supplementary Table S5). The results showed that during evolution, most NHX genes were strongly purified and selected, and only a few genes differentiated and produced new biological functions.

### Conserved Sequence and Gene Structure Analysis

**FIGURE 4:**
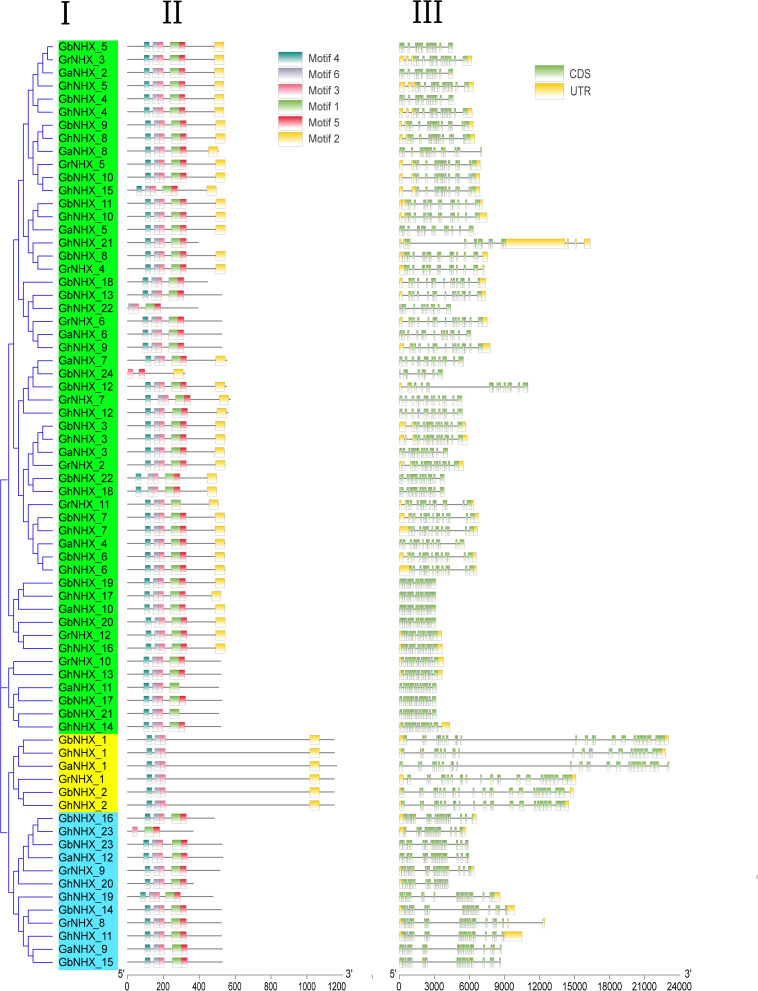
Gene structures and conserved protein motifs of NHXs. **(I)** NJ phylogenetic tree analysis of *G. hirsutum*. **(II)** Distributions of the predicted motifs in the NHX genes. **(III)** The numbers, lengths, and positions of exons and introns within NHX genes.

To understand the structural diversity and structural characteristics of NHX genes, motif analysis of 71 NHX amino acid sequences of four cotton genomes (*G. hirsutum, G. barbadense, G. arboreum*, and *G. raimondii*) was carried out by the MEME program (Figure 4). A total of 6 motifs were identified in the NHXs, and only motif 3 existed in all genes. Motifs 2, 3, 4, and 6 appeared in PM-class NHX genes. Most of the Endo-class genes contained motifs 1, 3, 4, 5, and 6, while *GhNHX_23* contained only motifs 1, 3, and 5. Of the genes in the Vac-class, 39 genes, 10 genes, 3 genes and 1 gene contained 6, 5, 4, and 3 motifs, respectively. Figure 4 shows that the numbers and types of motifs in the NHXs were significantly different between groups and were almost similar within groups, proving that the amino acid sequences and gene structures of NHX genes were highly conserved. These results serve as a basis for predicting the function of the NHX gene family.

The exons and introns of *G. hirsutum, G. barbadense, G. arboreum* and *G. raimondii* were analyzed and compared by the online program GSDS^1^ (Figure 4). The numbers of exons of *GhNHX_1, GhNHX_2, GaNHX_1, GbNHX_1, GbNHX_2*, and *GrNHX_1* located on the cell membrane were the highest (23–24). A total of 12 genes in the four species were located in the Endo-class, 11 of which had between 18 and 23 exons, and only *GrNHX_8* had 24 exons, while the number of exons of the other genes in the Vac-class varied between 8 and 16. The numbers of exons in the different classes were obviously different, ranking from lowest to highest as PM-class < Endo-class < Vac-class. The numbers of exons and introns between species were relatively conserved.

### Analysis of *Cis*-Acting Elements in NHX Promoter Regions

**FIGURE 5:**
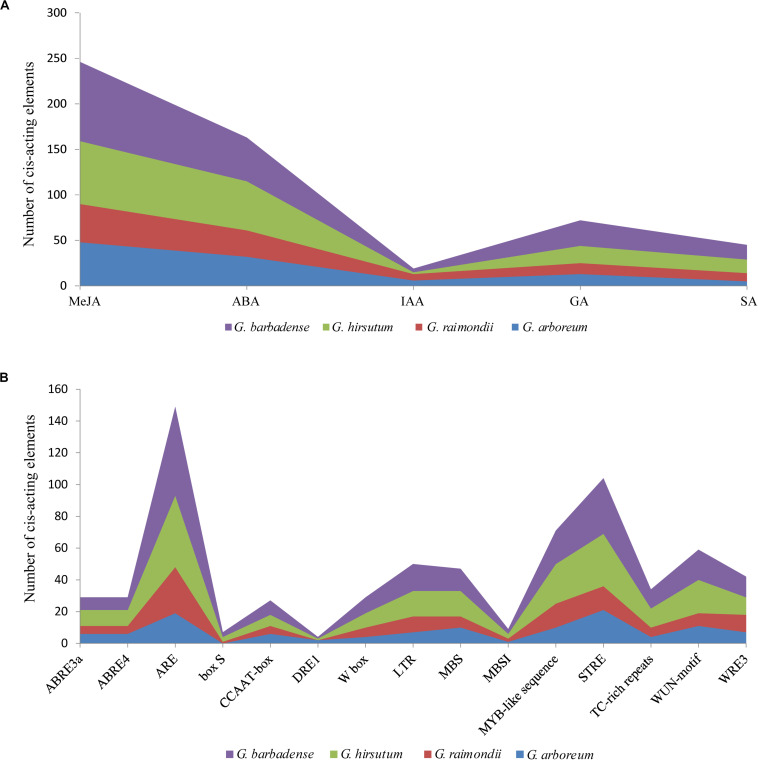
Distribution characteristics of phytohormone-responsive and stress-related *cis*-acting elements in NHX promoter fragments of four cotton species (*G. hirsutum, G. barbadense, G. arboreum* and *G. raimondii*). **(A)** The sum of five phytohormone-responsive cis-acting elements [methyl jasmonate (MeJA), abscisic acid (ABA), auxin (IAA), gibberellin (GA), and salicylic acid (SA)] in the NHX promoter fragments of four cotton species. **(B)** The number of *cis*-acting elements related to stress in the four cotton species.

To better study gene expression and transcriptional regulation, 71 NHX promoter sequences of the four cotton species were identified by PlantCARE software (Figure 5 and Supplementary Table S6), and mainly stress- and hormone-related *cis*-acting elements were analyzed. Among the identified cis-acting elements, 545 were related to hormones, including ABA, salicylic acid (SA), auxin (IAA), gibberellin (GA) and jasmonate. Of these, ABA (163) and methyl jasmonate (MeJA) (246) were the largest types, accounting for 29.9 and 45% of all identified *cis*-acting elements of hormones, respectively. A total of 690 *cis*-acting elements were related to stress, such as low temperatures, drought, trauma, and anaerobic conditions. Among these elements, the contents of antioxidant- and defense-related reaction elements were higher. In addition, among the four cotton species of *G. hirsutum, G. barbadense, G. arboreum*, and *G. raimondii*, W BOX (a salt-responsive element) predicted 9, 10, 4, and 6 elements, respectively (Figure 5B and Supplementary Table S6). The results of these *cis*-acting elements suggest that NHXs may play an important role in hormone regulation and stress responses in cotton.

### Expression Characterization of GhNHXs in Different Tissues

**FIGURE 6:**
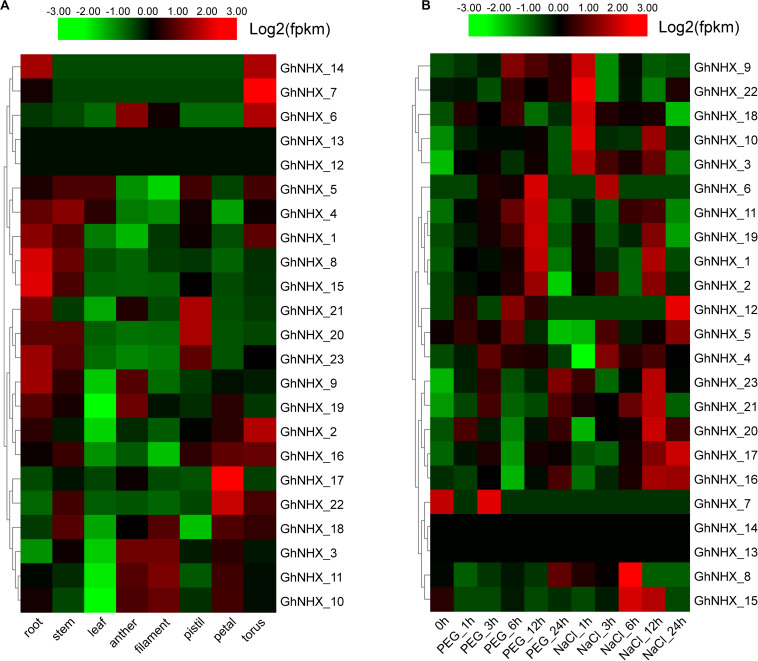
Expression profiles of GhNHXs in different tissues **(A)** and responses to different stresses **(B)**. The tissues or treatments are shown at the bottom, genes are shown on the right, and the phylogenetic relationships are shown on the left.

To study the biological function of GhNHXs in different tissues, we analyzed spatio-temporal expression patterns in root, stem, leaf, anther, filament, pistil, petal, and torus tissues from transcriptome data (Figure 6A). The Figure 6A showed that 2 genes (*GhNHX_12* and *GhNHX_13*) had low or no expression in the 8 tissues. *GhNHX_20* and *GhNHX_21* were predominantly expressed in pistil; *GhNHX_17* and *GhNHX_22* were predominantly expressed in petal; *GhNHX_2* and *GhNHX_7* were predominantly expressed in torus. *GhNHX_1, GhNHX_4, GhNHX_8, GhNHX_9, GhNHX_15*, and *GhNHX_23* were highly expressed in root and stem; *GhNHX_3, GhNHX_10, GhNHX_11*, and *GhNHX_18* were highly expressed in anther, filament or petal.

Clustering analysis of the 23 GhNHX genes treated with polyethylene glycol (PEG) or NaCl (Figure 6B) showed that the expression patterns in transcriptome data from the two treatment conditions were different. In PEG treatment, the expression of *GhNHX_1, GhNHX_2, GhNHX_6, GhNHX_11*, and *GhNHX_19* reached the highest level at 12 h after treatment. In salt treatment, the expression of *GhNHX_16* and *GhNHX_17* showed an overall upward trend from 0 h to 24 h. And *GhNHX_3, GhNHX_9, GhNHX_10, GhNHX_18* and *GhNHX_22* responded quickly to salt stress at 1 h. The expression of the *GhNHX_1, GhNHX_2, GhNHX_8, GhNHX_11, GhNHX_15, GhNHX_16, GhNHX_17, GhNHX_19, GhNHX_20, GhNHX_21*, and *GhNHX_23* were high at 6 or 12 h. At the same time, the Figure 6B also suggested that 4 genes (*GhNHX_1, GhNHX_2, GhNHX_6, and GhNHX_19*) showed obvious responses under PEG and NaCl treatments, indicating that these genes may be regulated by drought and salt stresses.

**FIGURE 7:**
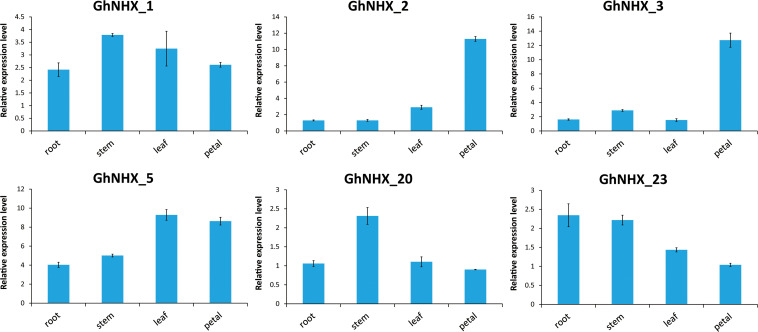
Expression analysis of GhNHXs in different tissues. Error bars show the standard deviation of three biological replicates.

According to the transcriptome data, six genes were selected for qRT-PCR analysis (Figure 7). *GhNHX_1* and *GhNHX_2* belong to the PM-class, *GhNHX_20* and *GhNHX_23* belong to the Endo-class, and *GhNHX_3* and *GhNHX_5* belong to the Vac-class. The results of qRT-PCR in 4 tissues showed that *GhNHX_2* and *GhNHX_3* were highly expressed in petal; *GhNHX_1* and *GhNHX_20* were more expressed in stem; *GhNHX_5* was more expressed in leaf and petal; *GhNHX_23* was more expressed in root and stem. The results showed that GhNHXs were expressed in different tissues.

### Expression Analysis of GhNHX Genes Under Salt Stress

By analyzing the *cis*-acting elements of the promoter, we speculated that GhNHXs may be related to the stress response. According to the analysis of transcriptome data, some genes have relatively high expression under different stresses (Figure 6B). Most genes responded to NaCl stress, and different genes had different responses to different treatment times. For example, the expression of *GhNHX_3* and *GhNHX_18* was relatively high under NaCl stress for 1 h and decreased gradually with increasing treatment time, and the expression of *GhNHX_16* and *GhNHX_17* gradually increased with increasing treatment time. In addition to the response to salt treatment, a response to PEG treatment was also observed. These results showed that GhNHXs can improve resistance to abiotic stress through different expression patterns in response to stress and provide candidate genes for further investigation of salt stress.

**FIGURE 8:**
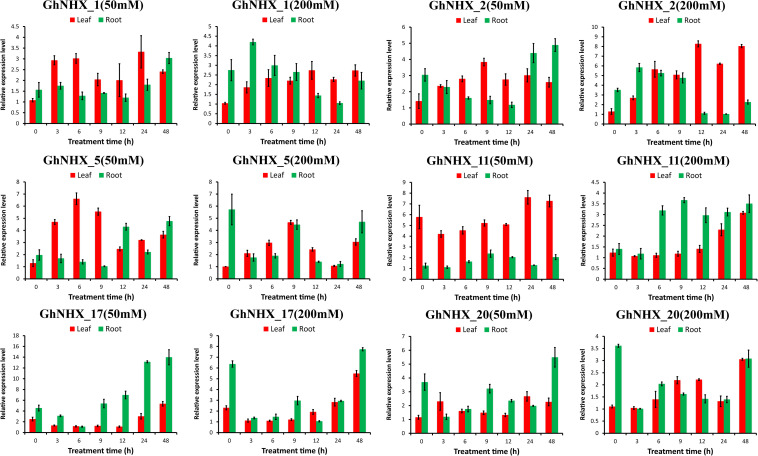
Relative expression levels of GhNHX genes in the leaves and roots of *G. hirsutum* TM-1 seedlings subjected to 50 and 200 mM NaCl for 0, 3, 6, 9, 12, 24, and 48 h. The 2^–ΔΔ*Ct*^ method was used to calculate the expression levels of GhNHX genes at different times. Error bars show the standard deviation of three biological replicates.

To further analyze the response of GhNHXs to salt stress, *GhNHX_11* and *GhNHX_20* (Endo-class), *GhNHX_1* and *GhNHX_2* (PM-class), and *GhNHX_5* and *GhNHX_17* (Vac-class) were selected for verifying by qRT-PCR. As shown in Figure 8, GhNHX genes were induced during salt treatment. Under the same salt concentration, the expression trends in roots and leaves were different. Under low-salt treatment (50 mM NaCl), the expression of *GhNHX_2* and *GhNHX_5* increased at first and then decreased in leaves, and the expressions of *GhNHX_1, GhNHX_2, GhNHX_5, GhNHX_17*, and *GhNHX_20* were the highest at 48 h in roots. Under high-salt stress (200 mM NaCl), the expressions of *GhNHX_1, GhNHX_2, GhNHX_11, GhNHX_17*, and *GhNHX_20* reached the maximum at the later stage of treatment (12–48 h), and the expressions of *GhNHX_11* and *GhNHX_17* increased gradually with the extension of treatment time in leaves. In roots, the expressions of *GhNHX_1* and *GhNHX_2* were higher at 3 h and lower at 24 h, and the expressions of *GhNHX_5* and *GhNHX_17* were higher at 9 and 48 h. The expression of *GhNHX_11* maintained a high level from 6 to 48 h. These results suggested that these genes might play an important role in response to salt stress.

### Responses of GhNHX Genes to MeJA and ABA Treatment

**FIGURE 9:**
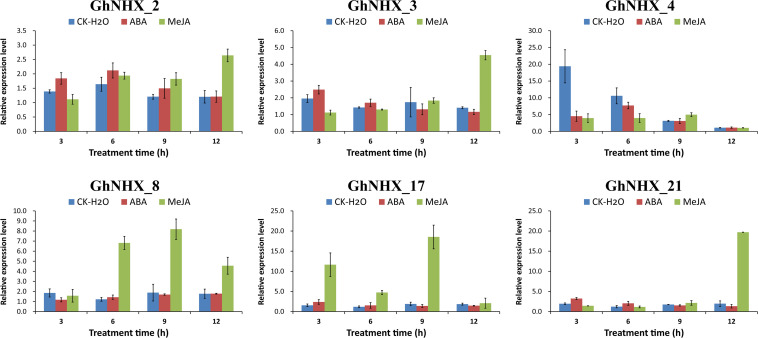
Relative expression levels of GhNHX genes under ABA and MeJA treatments. Two true leaves were sprayed with 200 mM ABA (red) and 100 mM MeJA (green), and water (blue) was used as a blank control. Error bars show the standard deviation of three biological replicates.

Among the predicted promoters, the contents of MeJA and ABA hormone response elements were found to be the highest in the GhNHX gene family (Figure 5A). To further explore the effect of hormones on the NHX family, 6 GhNHX genes containing these two hormone response elements were selected for qRT-PCR analysis. The results showed that after MeJA hormone treatment, compared with the control at the same time point, all 6 genes reached maximum levels at the time point after MeJA treatment, 3 of which (*GhNHX_2, GhNHX_3*, and *GhNHX_21*) showed a trend of continuous upregulation (Figure 9), reaching maximum levels at 12 h, while the other three genes (*GhNHX_4, GhNHX_8*, and *GhNHX_17*) reached maximum levels at 9 h. The results from ABA treatment showed that *GhNHX_2* and *GhNHX_4* reached maximum levels at 6 h after treatment, *GhNHX_3* and *GhNHX_21* decreased slightly, and *GhNHX_8* showed an upward trend after treatment (Figure 9). These results suggest that the transcriptional levels of GhNHXs may be closely related to the regulation of MeJA and ABA.

## Discussion

The NHX family plays an important role in plant cells and is indispensable for normal life activities. NHX proteins can help absorb K^+^ ions under normal conditions and can be stored in vacuoles for cellular storage, turgor generation and pH regulation. Under salt or osmotic stress, NHX proteins fulfill a protective function through vacuolar compartmentalization of K^+^ as well as Na^+^ in some cases, thereby preventing toxic Na^+^-K^+^ ratios in the cytosol while accruing solutes to balance both (Jiang et al., 2010).

### Gene Expansion and Duplication of NHXs in the Evolution of Cotton

The production of polyploids is an expression of plant adaptability to the environment and an important mechanism for the formation of new species (Ramsey and Schemske, 1998). The replication of a single gene, chromosome or whole genome is the main force of plant genome evolution (Paterson et al., 2012). Data indicate an origin for *Gossypium* 5–15 million years ago (mya) and rapid early diversification of the major genome groups (Wendel and Cronn, 2003). Allotetraploid cotton (*G. hirsutum*) is the result of genomic hybridization and doubling approximately 1–1.5 mya (Ks peaks at 0.005 and 0.008, respectively) from diploid A (*G. arboreum*) originating in Africa and diploid D (*G. raimondii*) originating in Mexico (Wendel and Cronn, 2003; Li et al., 2015; Zhang et al., 2015), and gene loss is most likely an ongoing process in allotetraploid cotton (Zhang et al., 2015), which may have resulted in *G. hirsutum* (23) lacking an NHX gene compared with *G. barbadense* (24) and provides strong support for the study of cotton polyploidy. In this research, 23, 24, 12, and 12 NHX genes were identified in *G. hirsutum*, *G. barbadense*, *G. arboreum* and *G. raimondii*, respectively. The results showed that the number of NHXs in two tetraploid cotton species was almost the sum of the NHXs in two diploid cotton species, which resulted in more gene duplication in the tetraploid NHX gene family than in diploid cotton species, and the tetraploid genome was further verified to have been formed by doubling of the chromosomes of the two diploid genomes (Paterson et al., 2012). Although more NHXs were present in tetraploid cotton species than in diploid cotton species, genomic arrangements and chromosome doubling may have also led to some gene loss in the process of doubling from diploid to tetraploid cotton species (Paterson et al., 2012). The duplication types of 47 genes in *G. hirsutum* and *G. barbadense* showed that 46 NHX genes were formed by WGD or segmental duplication based on collinearity analysis (Supplementary Table S4). Additionally, cotton MADS-Box, the GT47 family and soybean WRKY showed enlargement as the result of WGD and segmental duplication (Yin et al., 2013; Ren et al., 2017; Wu A. et al., 2019), indicating that gene duplication plays an important role in the evolution of gene families (Cannon et al., 2004). The collinearity analysis of NHXs showed that the A genome and D genome of NHXs were similar to the At subgenome and Dt subgenome, respectively (Figure 3), indicating that they share the same ancestor (Qanmber et al., 2019). In conclusion, WGD or segmental duplication should be the main driving force for the NHX family from the diploid genome to the tetraploid genome, and these findings also provide a theoretical reference for the study of gene duplication, genetic information transmission between species and species evolution.

### Evolutionary Conservation and Divergence of NHX Genes

In this study, 8, 27, 23, 24, 12, and 12 NHXs were identified in the genomes of *Arabidopsis*, *P. trichocarpa*, *G. hirsutum*, *G. barbadense*, *G. arboreum*, and *G. raimondii*, respectively. The differences in the number of NHX genes in plants may be attributed to gene duplication and loss specific to different subfamilies of the NHX gene over the course of evolution (Wu G. Q. et al., 2019). In *Arabidopsis*, 8 NHXs were divided into three groups according to subcellular location, including 2 PM-class genes, 2 Endo-class genes and 4 Vac-class genes (Brett et al., 2005). In our research, 106 NHX genes from 6 species were also classified into 3 categories through phylogenetic tree analysis (Figure 2). Among them, the numbers of GhNHXs in these categories were 17, 2, and 4, respectively, and the number of genes in the Vac-class was the largest, indicating that NHXs in the Vac-class may play an important role in cells. Conservation of the gene sequence structure is the basis of the conservation of biological function. In this study, to explore the characteristics of NHX gene sequences in cotton, 71 NHX genes in 4 cotton species were analyzed for exon/intron structures and motifs (Figure 4), and the results showed that the structures and motifs of the NHX genes were similar. In *G. hirsutum*, the numbers of exons of *GhNHX_1* and *GhNHX_2* in class II (PM-class) were the largest, the numbers of exons of *GhNHX_23, GhNHX_20, GhNHX_19*, and *GhNHX_11* were the second largest, and the numbers of exons of *GhNHX_18, GhNHX_21* and *GhNHX_23* were the lowest, all of which were less than or equal to 16. The numbers of exons of the three NHX types (PM-class, Endo-class and Vac-class) in poplar were 23, 22, and 14, respectively (Tian et al., 2017). The largest number of exons in sugar beet was found in *BvNHX4* (Endo-class), with 26 exons, followed by *BvNHX5* (PM-class) with 23 exons (Wu G. Q. et al., 2019). In summary, structural similarities and quantitative diversity exist in exons and introns among different species, not only indicating that the NHX gene family conserved its gene function but also reflecting the diversity of selection in the process of evolution.

Previous studies have shown that most of the N-terminal domains of NHX proteins contain 9–12 TMs, which are highly conserved (Yamaguchi et al., 2003; Wu G. Q. et al., 2019). The domain composed of the amino acid sequence FFIYLLPPI was called the amiloride-binding site, which was sensitive to the Vac-class membrane NHX in the presence of the drug amiloride or its derivatives and was responsible for transport (Wu et al., 2011). In this study, 12 GhNHXs had this binding site in *G. hirsutum*, almost all of which were in the N-terminal TM3 of the Vac-class and 9–12 transmembrane fragments. The same results were also observed in *AtNHX1-4* of Arabidopsis (Aharon et al., 2003) and *ZxNHX1* of Zanthoxylum (Wu et al., 2011). These results indicated that the NHX reverse transporters of the Vac-class were highly conserved during evolution. In this study, through analysis of selective stress in the evolution of the NHX gene family, the results showed that the Ka/Ks ratio was less than 1 (Supplementary Table S5), indicating that the NHX gene family was strongly purified and selected during long-term evolution and is functionally conserved.

### *Cis*-Acting Elements of NHX in Upland Cotton

In eukaryotes, transcriptional regulation is the main mechanism of plant gene expression regulation, and *cis*-acting elements are involved in the transcriptional regulation of genes and play an important role in various biological processes, such as hormonal responses, abiotic stress responses and development (Ding et al., 2018; Verma et al., 2019; Wu A. et al., 2019). Different *cis*-acting elements have particularities and consistency in the promoter. The *cis*-acting element containing the AuxRE and DR5 motifs is usually an auxin-induced promoter (Ulmasov et al., 1997), light-induced promoters usually contain G-box, AT-rich, GT1-motif and I-box elements (Gilmartin et al., 1992; Foster et al., 1994), and the promoter containing CATGTG and CACG cis-acting elements is drought-induced (Tran et al., 2004). In plants, hormones such as ABA, ethylene, SA and IAA play important roles in plant growth and development and responses to stress (Díaz et al., 2002; Mishra et al., 2014; Liu et al., 2015; Shu et al., 2019; Wang and Huang, 2019). In this study, 71 NHX promoter fragments from four cotton species were extracted, and cis-acting elements were identified. We mainly statistically analyzed stress- and hormone-related *cis*-acting elements, as shown in Figure 5. The results showed that the *cis*-acting elements related to hormone responses were IAA, GA, SA, ABA, and MeJA; these five hormones existed in all four cotton species, and the contents of ABA and MeJA were the highest (Supplementary Table S6). qRT-PCR analysis revealed that exogenous application of MeJA and ABA had different effects on the induction of GhNHX gene transcription, especially exogenous MeJA treatment, and six genes were upregulated to different degrees and reached maximum levels at different time points (Figure 9). NHXs have been suggested to be regulated by many hormones, and ABA and MeJA hormones play more extensive roles in hormone regulation. In addition, *cis*-acting elements related to stress responses were identified, mainly including responses to low temperature, drought, wounding, MYB binding and anoxia. The proportion of each *cis*-acting element in different cotton species was similar, as shown in Supplementary Table S6, indicating that the NHX family may have the same regulation mode in cotton (Wu A. et al., 2019). In addition, W BOX, a cis-acting element associated with development processes and the salt response (Xu et al., 2018; Wu G. Q. et al., 2019), was predicted in all four cotton species (*G. hirsutum, G. barbadense, G. arboreum*, and *G. raimondii*) (Figure 5B), which can be recognized by the family of WRKY transcription factors. The W BOX *cis*-element may have important reference value in salt stress in the NHX family. In summary, the NHX family may be involved in the regulation of a variety of hormones and stress responses. These *cis*-acting elements enable plants to rapidly respond to external biological and abiotic stresses and improve the resistance of plants to the outside world, facilitating further understanding of the related mechanism of *cis*-acting regulatory elements in the stress response.

### Expression Analysis of GhNHX Genes Under Multiple Stresses

One important method to alleviate salt stress in plants is to isolate Na^+^ into vacuoles to maintain Na^+^ homeostasis and reduce the toxicity of Na^+^ to the cytoplasm (Li et al., 2017; Wu, 2018). Under PEG treatment, the expression of *GhNHX_1, GhNHX_2, GhNHX_6, GhNHX_11*, and *GhNHX_19* reached the highest level at 12 h, and *GhNHX_1, GhNHX_2*, and *GhNHX_19* also responded to salt stress in varying degrees, demonstrating these genes may be associated with salt stress and drought stress. Some studies verified that ABA and MeJA were related to salt tolerance (Zhang et al., 2019; Zhao et al., 2019). Depending on the qRT-PCR results of exogenous MeJA and ABA treatment, it was showed that *GhNHX_3, GhNHX_8, GhNHX_17*, and *GhNHX_21* were highly expressed at one specific time after MeJA treatment; *GhNHX_4* was more expressed at 6 h, which was 7–8 times higher than that at 12 h after ABA treatment (Figure 9), illustrating these genes may respond to MeJA and ABA hormone regulation. In addition, we identified the relative expression of 6 GhNHXs under salt stress by qRT-PCR. Under high-salt conditions (200 mM NaCl), there were differences between roots and leaves in the expression trends of GhNHX genes. The expression of *GhNHX_1* and *GhNHX_2* (PM-class) increased gradually within 12 h of salt treatment in leaves and remained at a high level from 12 to 48 h, while the expression in roots reached the highest level at 3 h, decreased gradually, and then increased again at 48 h (Figure 8). Similar results were observed in the BvNHX genes of *sugar beet* (Wu G. Q. et al., 2019). Similarly, the expression of the wheat gene *TaNHX3* increased within 24 h in roots or leaves and decreased gradually within 48 h, and transgene analysis confirmed that *TaNHX3* has important functions in regulating the plant tolerance to salt stress (Lu et al., 2014). The expression of the *Porteresia coarctata* gene *PcNHX1* in roots increased gradually within 24 h of salt treatment and then decreased gradually from 36 to 48 h and transgenic tobacco *PcNHX1* seedlings show growth advantage in increasing NaCl (Jegadeeson et al., 2019). Interestingly, during root treatment (Figure 8), the expression of *GhNHX_5, GhNHX_17*, and *GhNHX_20* decreased rapidly from 0 to 3 h under high-salt conditions (200 mM NaCl) and increased with increasing salt treatment time in contrast to the relatively low expression at 0 h under low-salt conditions, which may be related to regulation of the salt tolerance of upland cotton TM-1 with different concentrations of salt treatment. Under low-salt conditions (50 mM NaCl), The expression level of *GhNHX_1, GhNHX_2, GhNHX_5, GhNHX_17*, and *GhNHX_20* was the highest at 48h. The expression of *BvNHX1* and *BvNHX3* in sugar beet were significantly upregulated in NaCl solutions of different concentrations within 48 h (Wu G. Q. et al., 2019). According to the above analysis results, it was found that these genes (*GhNHX_1, GhNHX_2, GhNHX_5, GhNHX_17, and GhNHX_20*) were closely related to the response to salt stress, but their regulatory mechanism needs to be further studied.

## Materials and Methods

### Identification and Characterization of the NHX Genes

Eight Arabidopsis ATNHX sequences were obtained from the *Arabidopsis thaliana* database TAIR (Supplementary Table S7) (Lamesch et al., 2012). The protein and genome sequences of *G. arboreum* (CRI, version 1.0), *G. raimondii* (JGI, version 2.1), *G. hirsutum* (HAU, version 1) and *G. barbadense* (HAU, version 1) were downloaded from the cotton genome database CottonFGD (Zhu et al., 2017). The protein and genome sequences of *P. trichocarpa* were downloaded from the NCBI database.^2^ The hidden Markov model (HMM) profile was constructed using eight ATNHX genes, and the NHX genes in four cotton species and *P. trichocarpa* were identified by HMMER 3.0 software with an *E* value threshold (E) < 10^–40^ (Potter et al., 2018). Furthermore, the conserved domains of all the candidate NHX protein sequences were identified using the online Simple Modular Architecture Research Tool (SMART) (Letunic et al., 2015).

Transmembrane domain analysis of NHX sequences was performed using TMHMM 2.0^3^ (Sonnhammer et al., 1998; Krogh et al., 2001). Phosphorylation sites were predicted using netphos 3.1 (Blom et al., 2004). The physicochemical properties of NHX proteins were analyzed by the ExPASy Proteomics Server (Bjellqvist et al., 1993).

### Phylogenetic Analysis

To study phylogenetic relationships among different species, multiple sequence alignments of NHX family proteins from several species, including *A. thaliana*, *P. trichocarpa*, *G. hirsutum*, *G. barbadense*, *G. arboreum*, and *G. raimondii*, were carried out using the ClustalW function of MEGA 7.0 software, and the NJ method was used to compare the results. The bootstrap method setting value for the phylogenetic tree was 1000, and the default values were used for other parameters (Kumar et al., 2016). Visualization of developmental trees was performed by iTOL^4^ (Letunic and Bork, 2016).

### Chromosome Distribution of NHX Genes and Analysis of the Ka/Ks Ratio

According to analysis of the cotton genome database, the physical locations of NHX genes in four cotton species [*G. hirsutum* (Gh), *G. barbadense* (Gb), *G. arboreum* (Ga), and *G. raimondii* (Gr)] were determined. Collinearity analysis of protein sequences of the four cotton species was performed using BLASTP and MCScanX software (Wang et al., 2012). TBtools software was used to visualize the chromosome location and collinearity results, and the NG function was used to estimate the Ks and Ka replacement rates (Yang and Nielsen, 2000; Chen et al., 2018).

### Analysis of Conserved Motifs, Gene Structures, and *Cis*-Acting Elements

The conserved domains of NHX genes were analyzed using the online software MEME5.1.0,^5^ the maximum number of motifs was set to 6, and other parameters were set to default values (Bailey et al., 2009).

GSDS was used to analyze the members of the gene family by comparing the CDS sequence of NHX genes with the corresponding genome sequence (Hu et al., 2015). CDS sequence and genome sequence data packages were downloaded from CottonFGD,^6^ and the result of the TBtools software analysis was used to visualize the image (Chen et al., 2018). The 2000-bp upstream regions from the initiation codons (ATG) of NHXs were analyzed by PlantCARE software, and the *cis*-acting elements in the promoter were determined (Lescot et al., 2002).

### Plant Materials and Treatments

The Upland Cotton Cultivar “Texas Marker-1” (TM-1) was planted in Anyang City, Henan Province, China. RNA was extracted from roots, stems, leaves and petals. After soaking TM-1 seeds in distilled water overnight, uniform seeds were transferred to plastic containers containing Hoagland nutrient solution and cultured in a greenhouse (light and dark cycle: 28°C for 16 h/22°C for 8 h) to study the reaction to NaCl treatment. When two true leaves were cultured, Hoagland nutrient solution containing 200 mM NaCl was used to treat them. Roots and leaves treated for 0, 3, 6, 9, 12, 24, and 48 h were used for RNA extraction.

Similarly, TM-1 seeds were planted in a greenhouse, and when they grew to two flat true leaves, they were sprayed with 100 mM MeJA or 200 mM ABA for treatment, and water was used as a control. Three leaves of seedlings were collected for RNA extraction at 3, 6, 9, and 12 h of treatment. All samples were immediately frozen in liquid nitrogen and stored at −80°C.

### Transcriptome Data Analysis and Quantitative Real-Time Polymerase Chain Reaction (qRT-PCR)

Raw RNA-seq data (SRA: PRJNA490626) were downloaded from the NCBI Sequence Read Archive. RNA-seq expression data were analyzed by TopHat2 (Kim et al., 2013) and cufflinks (Trapnell et al., 2010), and gene expression was measured in fragments per kilobase million (FPKM). A heat map was created by TBtools software. RNAprep Pure Plant Kit (TIANGEN, Beijing, China) was used to extract total RNA from collected samples. A PrimeScript RT Reagent kit (Takara, Japan) was used to synthesize cDNA. The sequences of qRT-PCR primers are shown in Supplementary Table S8. GhACTIN was used as a constituent expression control in qRT-PCR experiments. An ABI 7500 real-time PCR system (Applied Biosystems, United States) was used to perform qRT-PCR (Promega, Madison, WI, United States) with three biological replicates. The relative expression level of GhNHXs was calculated by the 2^–ΔΔ*CT*^ method.

## Conclusion

The NHX gene family plays an important role in the study of plant salt tolerance. In this study, 71 members of the NHX family from four cotton species were identified, and their gene sequences, phylogeny and biological characteristics were analyzed by comprehensive bioinformatics. The purpose of this study was to explore the conservative structures, evolutionary relationships and regulatory elements of the NHX family and finally reveal this family’s important role in the regulation of salt stress in cotton. Through qRT-PCR analysis, we found that the expression of some NHXs showed an upward trend in cotton under salt stress, and the expression patterns of some genes were different under low- and high-salt conditions. These results indicate that the NHX family plays an important role in the response of cotton to salt stress, and the systematic bioinformatics analysis also serves as a solid foundation for the exploration of cotton NHX genes.

## Data Availability Statement

Publicly available datasets were analyzed in this study. This data can be found here: SRA: PRJNA490626.

## Author Contributions

XF and HWa conceived and designed the study and prepared the manuscript. XF, ZL, JZ, XY, HWe, and MK performed the experiments. HWa, AW, LM, and JL assisted with the analysis and interpretation of the data. SY participated in the design of the experiments and provided a critical review. All authors contributed to the article and approved the submitted version.

## Conflict of Interest

The authors declare that the research was conducted in the absence of any commercial or financial relationships that could be construed as a potential conflict of interest.

## Footnotes

1 http://gsds.gao-lab.org/

2 https://www.ncbi.nlm.nih.gov/genome/?term=Populus+trichocarpa

3 http://www.cbs.dtu.dk/services/TMHMM/

4 https://itol.embl.de/index.shtml

5 http://meme-suite.org/tools/meme

6 https://cottonfgd.org/

## Supplementary Material

TABLE S1 Nomenclature and sequencing of NHX genes in *G. hirsutum, G. barbadense, G. raimondii G. arboreum*, and *P. trichocarpa*.

TABLE S2 Detailed physicochemical characteristics of NHX proteins of *G. hirsutum*.

TABLE S3 Phosphorylation sites of GhNHXs.

TABLE S4 Duplicate type of NHXs in *G. hirsutum* and G. *barbadense*.

TABLE S5 The Ka/Ks ratios of duplicated gene pairs.

TABLE S6 Analysis of cis-acting elements in NHX gene promoter regions.

TABLE S7 Eight Arabidopsis ATNHX sequences.

TABLE S8 Primer pairs used in the qRT-PCR analysis.
